# A case of novel mutation in *ANOS1* (*KAL1)* gene and review of Kallmann syndrome

**DOI:** 10.1530/EDM-22-0310

**Published:** 2023-06-09

**Authors:** Sumeet Arora, Olga Yeliosof, Vivian L Chin

**Affiliations:** 1 Department of Pediatrics, Division of Pediatric Endocrinology, Artemis Hospital, Gurgaon, Haryana, India; 2 Division of Pediatric Endocrinology, Cohen Children’s Northwell Health, Staten Island, New York, USA; 3 Division of Pediatric Endocrinology, SUNY Downstate Health Sciences University, New York, USA

**Keywords:** Adolescent/young adult, Male, Black - Caribbean, United States, Pituitary, Puberty, Insight into disease pathogenesis or mechanism of therapy, June, 2023

## Abstract

**Summary:**

Kallmann syndrome (KS) is a genetically heterogeneous condition characterized by hypogonadotropic hypogonadism with coexisting anosmia or hyposmia along with potential other phenotypic abnormalities depending on the specific genetic mutation involved. Several genetic mutations have been described to cause KS. The *ANOS1* (*KAL1)* gene is responsible for 8% of mutations causing KS. A 17-year-old male presented to our clinic with delayed puberty and hyposmia, along with a family history suggestive of hypogonadism in his maternal uncle. Genetic testing for KS revealed complete exon 3 deletion in the *ANOS1* gene. To the best of our knowledge, this specific mutation has not been previously described in the literature.

**Learning points:**

- Missense and frameshift mutations in the *KAL1* or *ANOS1* gene located in the X chromosome are responsible for 8% of all known genetic mutations of Kallmann syndrome.
- Exon 3 deletion is one of the *ANOS1* gene is a novel mutation, not reported before.
- Targeted gene sequencing for hypogonadotropic hypogonadism can be employed based on the phenotypic presentation.

## Introduction

Kallmann syndrome (KS) is a genetically heterogeneous disorder characterized by isolated hypogonadotropic hypogonadism (IHH) with anosmia or hyposmia, caused by defective migration of olfactory and gonadotropin-releasing hormone (GNRH) neurons from the olfactory placode region across the cribriform plate to the bulb (1). The prevalence has been estimated to be approximately 1 in 8000 males. In females, the prevalence is about five times less than that in males (2). KS may include phenotypic abnormalities including craniofacial defects (cleft lip, cleft palate, high-arched palate, ocular hypertelorism, dental agenesis), sensorineural deafness, digital anomalies (clinodactaly, syndactaly, camptodactaly), and neurological defects (oculomotor abnormalities, bimanual synkinesis or mirror hand movements, cerebellar ataxia). Normosmic IHH on the other hand is usually not associated with any other malformations (1).

IHH presents as gonadal failure due to inadequate production of GNRH or gonadotropins from the hypothalamus or pituitary, respectively. Children will typically present with partial or complete lack of pubertal development secondary to deficient GNRH-induced gonadotropin secretion with or without olfactory dysfunction.

Sporadic cases of IHH are the most frequent, and X-linked, autosomal dominant, and autosomal recessive patterns have all been reported. Mutations in several genes affecting GNRH neuronal migration have been identified in KS: *ANOS1* (Xp22.3), *FGF8* (10q24.3), *FGFR1* (8p11.2-p11.1), *PROK2 (*3p13), *PROKR2* (20p12.3), *NELF* (9q34.3), *CHD7* (8q12.1-q12.2), *WDR11* (10q26.12), *HS6ST1*(2q14.3), *SEMA3A* (7q21.11) (3).

We report a case of a 17-year-old male with delayed puberty due to IHH and hyposmia consistent with KS who was found to have a large deletion of exon 3 of the *ANOS1* gene. To the best of our knowledge, this is the first report of exon 3 deletion in the *ANOS1* gene as the cause of KS.

## Case presentation

A 17-year-old male was referred to the pediatric endocrinology clinic due to concerns about delayed puberty. His initial symptoms of adrenarche began at 13 years of age heralded by adult-type body odor, axillary hair followed by deepening of the voice without any facial hair. He reported occasional penile erections weekly. The mother initially denied any pertinent family history. There were no cognitive or behavioral concerns. He was born at 37 weeks via C-section due to breech presentation, one of twins with a birth weight of five pounds two ounces and no history of cryptorchidism. A review of systems was significant for polyuria, polydipsia, and weight gain with no neurological or hypogonadal symptoms. A decreased sense of smell with onset around 10 years of age and a history of symptoms of allergic rhinitis necessitated an ENT evaluation and antiallergic medications were initiated. Past history included tonsillectomy and adenoidectomy for obstructive sleep apnea at 15 years of age. His height was 166.6 cm (s.d. = −1.18, 12th percentile for sex and age) with a mid-parental sex-adjusted target height of 176.5 cm (s.d. = −0.4) and BMI above the 97th percentile. On examination, no dysmorphic features were noted (including no midline defects or digital anomalies). Acanthosis nigricans was noted on his neck. His nasal turbinates appeared erythematous. He was unable to correctly identify the odor on the smell test. Cranial nerves II–XII were intact with symmetric deep tendon reflexes bilaterally. There were no involuntary or mirror movements noted. His testes were bilaterally descended with the prepubertal volume of 3 mL each and pubic hair in Tanner 4 distribution. Measured stretched penile length was 6.5 cm which was expected for his Tanner 1 stage.

Differential diagnosis of constitutional delay of growth and puberty (CDGP) vs IHH more likely was considered.

## Investigations

Initial diagnostic evaluation is shown in Table 1. His growth factors were in accordance with his Tanner stage. Testosterone levels and gonadotropin levels were prepubertal. Bone age was significantly delayed. A1C level was in the prediabetic range. Testosterone replacement therapy was given to induce puberty. Testosterone enanthate intramuscular monthly injections were initiated at 50 mg monthly for the first 3 months, later increased to 75 mg monthly for a total duration of 6 months. Appropriate penile growth was achieved. The gonadotropins and testosterone remained prepubertal. MRI of the brain and pituitary gland showed no visualization of olfactory bulbs however imaging was suboptimal obscured by metallic artifact from the patient’s braces (Table 2).

**Table 1:** Initial laboratory evaluation at presentation.

Laboratory test	Reference values	Patient’s values^†^
LH, mIU/mL	0.02–0.3*	<0.10
FSH, mIU/mL	0.26–3.0*	0.37
Testosterone, ng/dL	<2.5–10*	2.5
IGF1, ng/dL	151–521	240
IGFBP3, µg/L	2657–6319	4747
TSH, µIU/dL	0.35–4.70	1.57
Free T_4_, ηg/dL	0.71–1.85	0.99
Prolactin, ηg/mL	0.0–15.0	2.7

*Prepubertal values; ^†^Age: 17 years.

FSH, follicle-stimulating hormone; LH, luteinizing hormone; IGF1, insulin-like growth factor 1; IGFBP3, insulin-like growth factor-binding protein 3; T_4_, thyroxine; TSH, thyroid-stimulating hormone.

**Table 2:** Imaging and genetic studies.

Study	Results
Bone age	Bone age of 14 years at chronological age 17 years (delayed bone age)
DXA	Normal for age, Z-score −0.2 at femoral neck, −1.4 lumbar spine, −0.4 at total hip.
Renal sonogram	No anatomical abnormality found
MRI brain and pituitary	Sella and pituitary slightly heterogeneous without focal finding, region obscured on T2 coronals. Normal in size and location. No marked or acute findings, images degraded by metallic artifact from braces.
Genetic testing	Complete lack of amplification of Exon 3 on *ANOS1* gene
	GeneDx (Gathersburg, MD, USA) hypogonadotropic hypogonadism panel/ sequencing and deletion/duplication analysis of 14 genes consisting of: *CHD7, FGF8, FGFR1, GNRH1, GNRHR, ANOS1, KISS1, KISS1R, NR0B1, NSMF, PROK2, PROKR2, TAC3, TACR3*.

## Outcome and follow-up

At the follow-up visit, the mother revealed that her maternal uncle was on testosterone therapy and also had a history of hyposmia and infertility, without any confirmed diagnosis. Genetic testing for KS showed a complete lack of amplification of exon 3 of *ANOS1* gene consistent with KS (Table 2). To the best of our knowledge, this is a novel deletion of the *ANOS1* gene as a cause of KS. Genetic testing was also obtained for the patient’s half-brother who was found to have a hemizygous pathogenic deletion of exon 3 at the *ANOS1* gene. Genetic testing for the fraternal twin sister was deferred until a later date in view of no clinical indication.

## Discussion

CDGP is the most common cause of pubertal delay; however, with certain clues in history and examination, the diagnosis of IHH becomes more likely. Our patient presented with IHH and a late revelation of significant family history, the genetic evaluation for causes of IHH was delayed. In the workup of IHH where anosmia is apparent, MRI may be helpful for the diagnosis of KS if there was absent or abnormal olfactory bulbs or sulci (1). However, normal olfactory bulbs can be present in up to 20% of patients (4). In our patient with partial absence of smell, his brain MRI was inconclusive regarding visualization of olfactory bulbs due to braces artifact. With a strong index of suspicion at a later time when family history was revealed, genetic evaluation for KS was obtained which revealed a deletion of exon 3 on *ANOS1* gene. Although missense variants in exon 3 and other in-frame deletion events have been reported in association with KS, deletion of exon 3 has not been reported in literature.

Pubertal delay is the most typical presentation of hypogonadotropic hypogonadism in adolescents defined as failure to achieve a testicular volume of 4 mL by age 14 in boys (5). The diagnosis of CDGP, a normal variant, is the most common cause of pubertal delay but needs to be followed over time. Red flags to evaluate for IHH include anosmia, micropenis, cryptorchidism, deafness, digital anomalies, and significant delay in puberal onset. An absence of minipuberty of infancy may help narrow the diagnosis toward IHH in an infant with micropenis or cryptorchidism.

Differentiating CDGP from IHH is challenging due to overlapping clinical presentations. There is considerable similarity in hormonal profile with prepubertal gonadotropins seen in both. There is no perfect diagnostic test to differentiate between the two (6). An inhibin B level of less than 35 pg/mL was shown to have a high predictive value approaching 93–100% for IHH in boys (6). Although basal gonadotropins maybe lower in adolescents with IHH; however, they do not allow for effective discrimination between CDGP and IHH. Similarly, stimulation testing with GNRH/GNRHa showed that up to 30% have LH responses indistinguishable from CDGP. Various studies have used different hCG stimulation testing protocols to differentiate CDGP and IHH making it difficult to compare, which have resulted in a positive predictive value ranging from 82 to 100% (6).

Idiopathic hypogonadotropic hypogonadism is broadly categorized into anosmic (or hyposmic) IHH which is more commonly referred to as KS and normosomic IHH (nIHH). KS is thought to be due to embryonic maldevelopment and migration of the GNRH neurons in association with olfactory neurons. Abnormalities in the GNRH neurons seated in the hypothalamus lead to nIHH. Certain genes may have an overlapping pathophysiological role making the differentiation between nIHH and KS blur. There are currently about 50 genes identified in IHH. The presence of specific anomalies and certain syndromic features may help narrow the differential and targeted genetic testing may be employed. Table 3 lists the genes associated with IHH.

**Table 3:** Genetic causes of idiopathic hypogonadotropic hypogonadism.

Developmental anomaly	Genes involved
GNRH pulse generator	*TAC3,TACR3, KISS1, KISS1R, GNRH1*
Kallmann syndrome (GNRH neuronal migration)	*ANOS1, FGFR1, FGF8, FGF 17, PROK2, PROKR2, HS6ST1, CHD7, SEMA3A, SEMA3E, IGSF10, SMCHD1, CCDC141, FEZF1, SMCHD1, WDR11, IL17RD, IGSF10, KLB, FLRT3, SPRY4*
Associated with hypothalamic–pituitary involvement	*NR0B1, HESX1, LHX3, PROP1, SOX2, NR5A1*
Isolated gonadotropic involvement	*FSHB, LHB, GnRHR*
Associated with other disorders	*DMXL2, RAB3GAP1/2, POLR3A, POLR3B* *PNPLA6, STUB1, OTUD4, LEP, LEPR, PC1*

Certain genes associated with KS will be discussed in this section.

Multiple genes have been associated with KS, but only 30% of KS have known gene mutations (7). KS is most commonly due to a sporadic mutation or inherited as familial (X-linked recessive, autosomal dominant with variable penetrance, or autosomal recessive). Known genes responsible for KS are summarized in Table 4.

**Table 4:** Genetic mutations associated with Kallmann syndrome (3, 10, 11).

Mutation	Location	Gene function and protein encoded	Phenotype (in addition to olfactory dysfunction and HH)
*ANOS1*	Xp22.3	Anosmin-1 promotes proper migration of GNRH neurons from olfactory epithelium to hypothalamus in fetal life	Bimanual synkinesis, renal agenesis, high-arched palate
*SEMA3A, SEMA3E*	7q21.11	Semaphorin 3A which is critical for GNRH neuronal migration; semaphorin 3E is involved in axonal growth	
*FGFR1*	8p11.2-p11.1	Fibroblast growth factor receptor 1 which interacts with anosmin-1 receptor	10% of cases (3); cleft lip/palate; digital bony abnormalities (polydactyly, syndactyly, and amptodactyly); dental agenesis; found in KS and normosmic IHH
FGF8	10q24.3	Fibroblast growth factor 8 which is a critical ligand for FGFR1 in GNRH ontogeny	10% of cases (3); cleft lip/palate; digital bony abnormalities (polydactyly, syndactyly, and amptodactyly); dental agenesis; found in KS and normosmic IHH
*PROK2*	3p13	Encodes ligand, prokineticin 2, and its receptor, prokineticin 2 receptor which play an essential role in morphogenesis of the olfactory bulb and GNRH secretion	Found in KS and normosmic IHH
*PROKR2*	20p12.3	Encodes ligand, prokineticin 2, and its receptor, prokineticin 2 receptor which play an essential role in morphogenesis of the olfactory bulb and GNRH secretion	Found in KS and normosmic IHH
*NELF*	9q34.3	Nasal embryonic LHRH-factor involved in GNRH migration	Found in KS and normosmic IHH
*CHD7*	8q12.1-q12.2	Chromodomain helicase DNA binding protein 7	Described in patients with CHARGE syndrome (coloboma, heart defects, choanal atresia, retardation, genital anomalies and ear anomalies; hearing impairment; cleft lip and cleft palate; found in KS and normosmic IHH
*WDR11*	10q26.12	WD repeat-contain protein 11 that interacts with EMX1, a homeobox transcription factor in specifying cell fats in central nervous system development as well as development of olfactory neurons	Found in KS and normosmic IHH
*HS6ST1*	2q14.3	Heparan sulfate 6-O-sulfotransferase 1 which is required for the function of FGFR1, FGF8 and ANOS1	Cleft lip/palate; limb deformities; found in KS and normosmic ISS
*FEZF1*	7q31.32	FEZ family zinc finger protein 1 is required for GNRH neuronal migration	
*IGSF10*	3q25.1	Immunoglobulin superfamily member 10 (IGSF10) is involved in neuronal migration	Maybe normosomic
*CCDC141*	2q31.2	Coiled-coil domain-containing protein 141 takes part in GNRH migration	An additional IHH gene variant identified in most patients with KS associated with *CCDC141* gene mutation.
*SMCHD1*	18p11.32	Encodes for epigenetic repressor in human olfactory epithelium.	Associated with congenital absence of nose along with cryptorchidism, microphallus in majority.

*ANOS1* gene is responsible for 8% of all cases of KS (2). *ANOS1* found on the X chromosome is responsible for this X-linked recessive condition, but an exon 3 deletion found in our patient has not yet been described in the literature. *ANOS1* encodes for extracellular matrix glycoprotein anosmin 1 which shares homology with molecules that are involved in GNRH neuronal migration and axonal path finding (8). Apart from its expression in the olfactory bulb and kidney, it is expressed in multiple other embryonic tissues. This gene comprises 14 exons on Xp22.3 chromosome. Loss of function mutations affecting the *ANOS1* gene caused by nonsense, frameshift mutations, splice site mutations, or large gene deletions have been described. Large gene deletions with the *ANOS1* gene from exons 4 to 14 have been reported before (9). Missense variants and other frameshift mutations in Exon 3 have been reported; however, to the best of our knowledge, the deletion of exon 3 has not been reported before. The phenotypic presentation of *ANOS1* gene mutation depends on the contiguous gene deletion. Most typical presentations involve olfactory dysfunction along with severe hypogonadotropic hypogonadism. Seventy-five percent may have bimanual synkinesis and 30% may have unilateral renal agenesis and high-arched palate (2).

Fibroblast growth factor 8 (*FGF8*) and fibroblast growth factor receptor 1 (*FGFR1*) mutations have an autosomal dominant inheritance with incomplete penetrance attributing to 10% of all KS cases (3). *FGFR1* encodes for tyrosine kinase cell surface receptors and plays a critical role in the olfactory system and GNRH ontogeny specification. *De novo* mutations in *FGFR1* are as high as 30%. The phenotypic presentation is variable from partial puberty to complete absence of puberty. *FGFR1* mutation has also been described in normosomic hypogonadotropic hypogonadism; 20% of patients with this mutation have demonstrated reversal of their hypogonadism. Non-reproductive phenotypes are also variable and include hearing loss, cleft lip or cleft palate, dental agenesis, camptodactyly or syndactyly, and hyperlaxity of digits (3, 10).

Prokineticins (PROK) are cysteine-rich proteins that are ligands to the G-protein coupled Prokineticin receptor (PROKR). PROK2 and PROKR2 specifically have an essential role in the morphogenesis of the olfactory bulb and GNRH secretion. *PROK2/PROKR2* mutations are responsible for 7% of KS thus far. The phenotypic presentation includes anosmia or hyposmia along with severe HH.

Other even rare genetic mutations causing KS include *CHD7, NELF*, *SEMA3A*, *WDR11*, and *HS6ST1* which contribute to only 1–6% of KS. *CHD7* mutations causing anosmia and hypogonadotropic hypogonadism (KS) have been described in patients with CHARGE syndrome (coloboma, heart defects, choanal atresia, retardation, genital anomalies, and ear anomalies)

Management of hypogonadism in KS should take into consideration the desire of fertility at the time of therapy and beyond. Testosterone replacement therapy is initiated to induce virilization and maintain secondary sexual characteristics as well as to preserve bone mass. Should fertility be desired, sperm retrieval can be done after administration of pulse GNRH therapy or combined FSH/hCG therapy to stimulate spermatogenesis, under the guidance of a fertility specialist (6).

Our patient was started on testosterone replacement therapy with IM testosterone enanthate and slowly titrated up to adult dose for development and maintenance of secondary sexual characteristics. He was eventually switched to Androderm® (testosterone transdermal) patches 4 mg daily and is doing well.

In conclusion, KS is a genetically heterogeneous condition caused by mutations in genes that control morphogenesis, migration, and survival of the olfactory placode and GNRH neurons. Variable presentation includes hypogonadotropic hypogonadism and anosmia or hyposmia. The *ANOS1* gene, located on the short arm of the X chromosome, is the most studied, and several missense, frameshift mutations, and exon deletions have been described in *ANOS1-*causing KS. Our patient has KS due to a novel mutation, a deletion of exon 3 on the *ANOS1* gene.

## Declaration of interest

The authors declare that there is no conflict of interest that could be perceived as prejudicing the impartiality of the research reported.

## Funding

This work did not receive any specific grant from any funding agency in the public, commercial or not-for-profit sector.

## Patient consent

Written informed consent for publication of their clinical details was obtained from the parent of the patient.

## Author contribution statement

Drs Arora, Yeliosof, and Chin conceptualized and prepared the manuscript. All authors have accepted responsibility for the entire content of this submitted manuscript and approved submission.
